# Expression of concern: Reduced miR-125a-5p level in non-small-cell lung cancer is associated with tumour progression

**DOI:** 10.1098/rsob.200151

**Published:** 2020-06-10

**Authors:** Hongxu Liu, Yegang Ma, Changhao Liu, Pengfei Li, Tao Yu

*Open Biol.* **8**, 180118. (Published online 10 October 2018) (doi:10.1098/rsob.180118)

Following the publication of ‘Reduced miR-125a-5p level in non-small-cell lung cancer is associated with tumour progression’ *Open Biol.* **8**: 180118. (Published online: 10 October 2018), the Editorial team have very serious concerns regarding the validity of the data used in this study.

We are issuing an expression of concern here and investigating this as a matter of urgency; we will notify readers as to the results of our investigation as soon as possible.

